# Supplementary material for: Characteristics and Correlation of the Microbial Communities and Flavor Compounds during the First Three Rounds of Fermentation in Chinese Sauce-Flavor Baijiu
Source: Foods. 2023 Jan 3;12(1):207. doi: 10.3390/foods12010207 (PMC9818360; doi:10.3390/foods12010207)
Supplement: Supplementary file 1 [file foods-12-00207-s001.zip › foods-2090300-supplementary.pdf]

Supplementary information for

Characteristics and Correlation of the Microbial Communities and Flavor Compounds during the First Three Rounds of Fermentation in Chinese Sauce-Flavor Baijiu

Youqiang Xu [1,2], Mengqin Wu [1], Jialiang Niu [1], Mengwei Lin [1], Hua Zhu [1,3], Kun Wang [1,3], Xiuting Li [1,2,*] and Baoguo Sun [1,2]

[1] Key Laboratory of Brewing Microbiology and Enzymatic Molecular Engineering of China General Chamber of Commence, Beijing Technology and Business University, Beijing 100048, China
[2] Beijing Advanced Innovation Center for Food Nutrition and Human Health, Beijing Technology and Business University, Beijing 100048, China
[3] Beijing Huadu Wine Food Limited Liability Company, Beijing 102212, China
* Correspondence: lixt@btbu.edu.cn

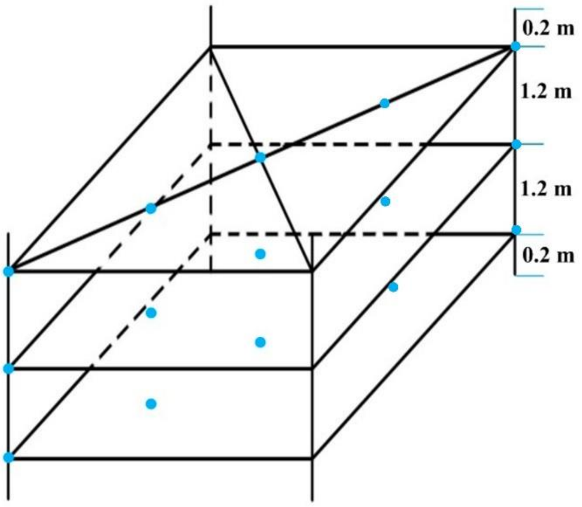

Figure S1. Sample collection positions of fermented grains in Jiaochi during sauce-flavor Baijiu manufacturing process.

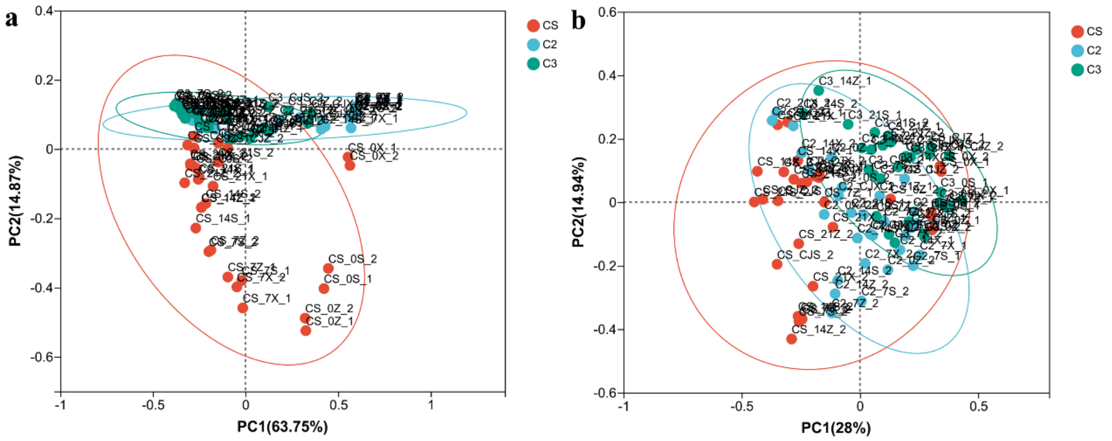

Figure S2. PCoA analysis of the bacterial genera (a) and fungal genera (b) of the fermented grain samples in rounds 1b, 2 and 3 of sauce-flavor Baijiu fermentation.

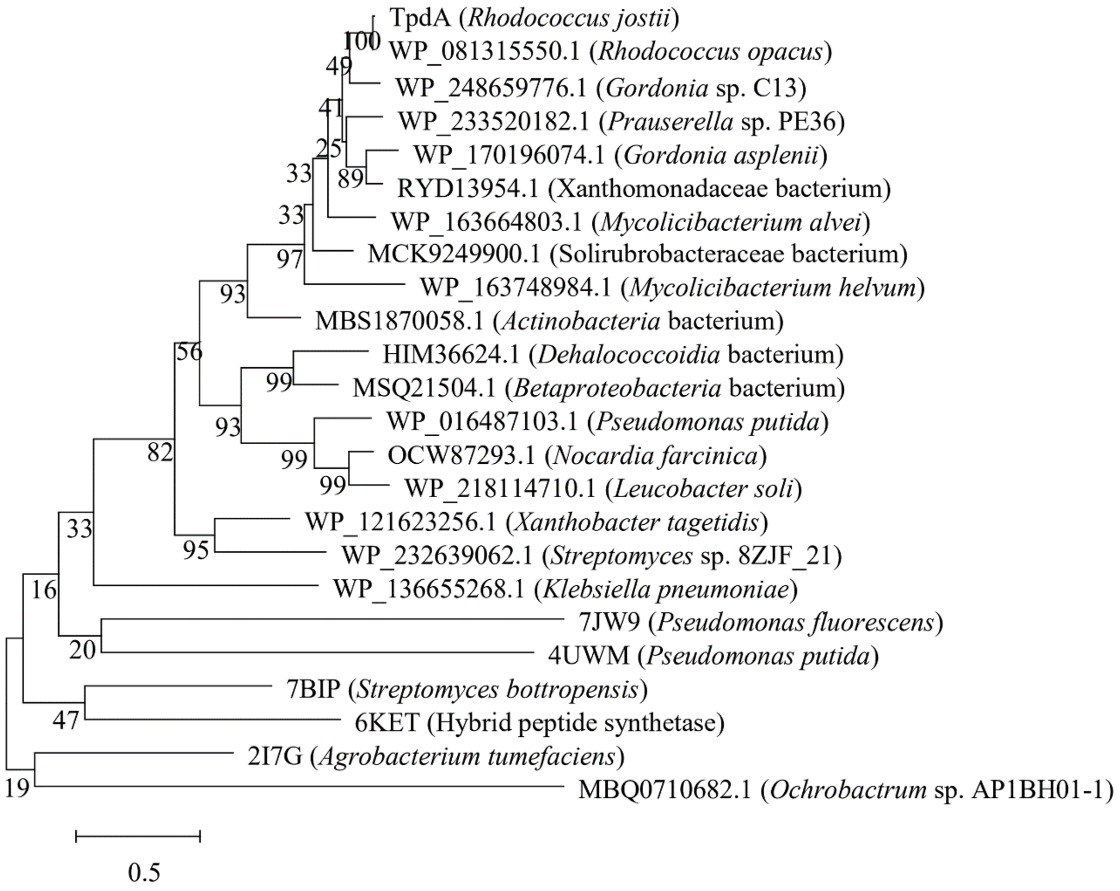

Figure S3. Evolutionary relationship analysis of tetramethylpyrazine oxygenase with homologous enzymes from different microorganisms by MEGA software with the Maximun likelihood method and a bootstrap value of 1000.

Table S1. Volatile compounds of the fermented grain samples from round 1b based on gas chromatography-mass spectrometry analysis (μg/kg).

Flavor compounds	CS_0S	CS_0Z	CS_0X	CS_7S	CS_7Z	CS_7X	CS_14S	CS_14Z	CS_14X	CS_21S	CS_21Z	CS_21X	CS_CJS	CS_CJZ	CS_CJX
Benzyl alcohol	10.34±2.12	17.52±5.91	10.53±1.28	44.91±6.63	23.68±6.28	18.29±3.93	41.07±17.07	40.71±12.7	ND	17.2±2.22	26.4±6.56	50.44±15.2	ND	ND	ND
Benzaldehyde	ND	ND	ND	ND	ND	ND	74.8±72.05	ND	ND	ND	35.51±10.08	66.97±26.73	20.64±2.36	24.34±4.94	37.69±14.04
Phenylacetic acid, ethyl ester	23.55±23.19	38.55±12.02	30.05±2.93	158.83±26.87	94.92±19.62	62.93±9.79	218.92±60.87	169.77±35.77	104.19±4.46	174.02±18.94	215.02±41.54	347.66±117.8	107.45±10.25	137.9±33.6	173.33±26.41
Acetic acid 2-phenylethyl ester	17.91±5.51	22.09±8.56	15.07±2.08	174±29.44	77.82±15.06	49.86±10	245.6±72.34	166.02±34.14	102.96±5.4	196.18±21.14	227.36±39.63	289.63±97.66	121.89±12.68	152.84±36.28	177.12±49
Pentanoic acid, 2-hydroxy-4-methyl-,ethyl ester	8.18±1.84	25.65±23.89	13.36±1.39	70.5±10.68	49.34±15.96	29.2±6.74	118.88±35.9	96.57±24.5	43.15±3.22	84.28±10.45	94.38±20.92	185.74±55.63	ND	ND	ND
Butanoic acid, ethyl ester	ND	ND	ND	ND	ND	12.71±2.61	31.38±28.57	22.63±19.65	32.01±1.76	42.15±4.9	71.57±14.28	156.2±29.76	23.81±0.25	36.58±10.86	75.66±10.62
Octanoic acid, ethyl ester	21.81±2.61	20.69±6.79	11.75±4.94	109.01±19.37	72.45±16.67	56.92±2.61	143.07±53.2	98.46±5.02	115.35±15.21	141.79±11.03	175.94±26.77	224.03±93.46	107.24±4.78	162.58±49.05	186.52±56.89
Dodecanoic acid, ethyl ester	8.83±4.36	ND	ND	49.7±7.6	40.4±5.46	27.49±1.58	70.99±19.26	55.71±8.47	44.59±3	60.96±10.11	74.61±15.63	92.18±23.92	48.84±2.4	68.01±16.61	100.42±73.13
(Z)-Ethyl heptadec-9-enoate	16.33±7.03	17.13±7.52	16.11±1.26	34.28±6.49	ND	ND	ND	ND	23.25±4.24	49.11±6.23	ND	ND	10.07±2.34	10.31±4.5	20.11±20.87
Decanoic acid, ethyl ester	27.01±7.01	14.67±13.53	ND	126.99±11.91	83.55±18.7	56.6±6.75	159.72±56.56	124.13±20.28	ND	ND	ND	ND	45.63±29.82	24.75±25.15	73.76±28.02
1-Hexanol	6.91±1.51	10.43±5.18	6.08±5.5	26.09±2.16	13.8±3.56	10.23±2.3	25.44±6.54	21.99±5.35	11.4±0.73	18.46±1.66	21.78±5.12	33.6±10.71	10.65±1.26	13.09±3.41	13.35±2.69
1-Octanol	8.81±1	ND	5.77±5	ND	ND	ND	20.06±17.47	ND	ND	ND	ND	ND	ND	ND	ND
Tetramethylpyrazine	14.15±6.66	24.24±9.12	29.45±6.91	59.53±23.57	23.68±5.74	17.55±3.19	33.03±34.98	37.64±9.08	15.5±1.61	22.57±2.11	28.18±5.52	63.88±14.7	ND	ND	ND
4-Ethylphenol	20.54±5.28	23.22±8.16	21.48±18.62	74.32±11.29	39.16±6.8	29.11±5.67	74.01±27.76	67.98±19.09	40.27±4.71	57.99±6.79	77.61±15.15	145.97±46.96	17.42±1.99	25.1±7.32	36.43±4.63
Butanedioic acid, diethyl ester	7.03±3.16	10.86±4.73	8.93±1.32	95.37±14.61	57.71±16.1	28.25±5.5	115.32±41.19	90.68±24.8	30.74±2.5	56.22±6.61	67.11±14.4	110.21±36.51	36.87±0.29	45±12.51	51.25±9.36
Nonanoic acid, ethyl ester	ND	ND	ND	ND	54.6±15.39	33.52±4.07	110.65±35.08	ND	49.73±5.36	72.15±7.13	80.6±15.14	66.95±83.26	22.79±1.13	28.71±7.98	33.68±15.46
3-Methyl-1-butanol	69.15±60.8	83.24±75.57	ND	960.8±204	413.31±103.91	268.85±58.02	805.84±302.66	690.74±204.18	261.84±20.13	508.62±65.4	520.26±118.16	901.68±271.34	198.29±26.11	253.2±64.5	306.02±40.74
Hexanoic acid, ethyl ester	8.41±7.3	11.1±2.53	ND	44.83±8.23	19.62±4.8	24.63±4.55	75.82±37.36	40.19±12.89	71.81±7.46	106.99±12.48	107.02±12.98	158.95±54.82	77.37±8.62	89.51±21.71	94.13±16.46
Acetic acid isoamyl ester	ND	6.17±5.52	ND	ND	31.35±4.52	41.17±8.28	129.03±59.96	76.41±24.11	86.66±11.77	211.72±15.29	208.42±33.99	261.3±82.53	ND	ND	ND

Tetradecanoic acid, ethyl ester	27.5±21.56	23.61±4.69	18.49±2.64	139.79±14.61	124.41±18.86	68.93±7.15	232.12±53.1	183.21±137.5	96.16±10.22	143.23±15.9	223.9±41.35	376.76±156.4	94.34±5.92	144.37±34.81	479.79±527.73
Nonanal	26.11±5.4	27.15±6.89	27.8±5.08	46.11±1.68	27.39±8.18	19.65±3.32	31.13±7.68	42.92±17.67	29.34±11.42	39.89±19.11	39.56±12.2	58.16±7.02	ND	ND	ND
Heptadecanoic acid, ethyl ester	ND	ND	ND	32.49±29.93	21.06±1.69	8.16±1.21	57.57±31.46	33.51±8.39	ND	23.71±3.49	63.8±12.58	43.08±13.8	8.33±1.45	15.04±5.34	23.6±20.47
Acetic acid ethyl ester	98.98±21.54	179.55±100.27	85.23±6.69	563.85±223.76	477.85±150.03	244.06±22.83	906.74±264.72	721.62±268.51	242.7±23.51	630.4±45.89	624.07±187.73	924.66±243.27	263.62±104.59	336.19±175.04	569.36±141.72
1-Nonanol	11.64±3.36	ND	9.29±0.78	ND	22.75±5.8	15.96±2.55	39.02±9.91	30.84±4.63	18±1.44	ND	22.48±2.09	25.77±27.14	17.47±1.41	18.73±4.16	ND
2,3,5-Trimethylpyrazine	2.94±2.55	9.43±4.24	11.92±1.95	49.5±44.18	ND	6.34±1.26	ND	ND	ND	ND	ND	ND	ND	ND	ND
2,4-Dimethylbenzaldehyde	5.44±4.71	7.02±6.96	ND	33.37±4.79	ND	ND	ND	ND	ND	ND	ND	ND	ND	ND	ND
Phenol,4-sec-butyl-2,6-di-tert	5.75±3.77	4.96±4.59	ND	ND	ND	ND	ND	ND	ND	ND	ND	ND	ND	ND	ND
Lactic acid isopentyl ester	ND	ND	ND	31.53±4.85	ND	11.52±3.88	46.38±15.07	33.79±8.84	15.28±2	30.28±4.41	35.18±8.74	38.92±35.05	ND	ND	29.38±32.53
Benzenepropanoic acid, ethyl ester	24.24±10.08	8.21±9.68	134.87±209.18	48.75±8.17	23.81±4.36	14.03±2.12	52.14±14.67	40.83±8.72	ND	46.13±6.55	49.88±10.01	71.25±26.56	26.89±2.59	30.48±7.18	35.68±6.4
Hexadecanoic acid, propyl ester	ND	ND	ND	ND	ND	ND	ND	ND	ND	18.57±2.6	24.99±3.94	52.4±21.38	ND	10.37±4.21	11.79±4.48
2,3-Butanediol	9.99±2.77	11.73±4.1	33.87±16.41	74.19±44	23.94±11.1	13.45±4.17	49.48±17.12	53.9±18.53	12.22±2.4	21.14±3.27	21.5±6.63	51.71±4.99	24.12±8.38	39.76±15.07	ND
4-Ethyl-2-methoxyphenol	49.85±19.51	59.3±15	34.44±4.58	221.67±44.84	100.61±16.84	82.33±15.89	235.05±84.38	190.2±50.61	108.05±14.72	123.05±17.09	193.87±38.6	367.17±83.01	ND	ND	ND
(Z)-Ethyl cinnamate	ND	ND	ND	ND	ND	ND	ND	ND	10.34±0.62	15.24±1.07	19±4.12	32.57±10.81	ND	ND	ND
3-Furaldehyde	20.55±5.25	28.63±12.06	ND	ND	ND	ND	ND	ND	ND	ND	ND	ND	ND	ND	ND
9-Hexadecenoic acid, ethyl ester	48.09±32.21	70.13±24.67	48.74±10.52	150.64±90.7	201.76±18.14	90.17±10.44	329.51±162.46	321.59±153.04	115.35±12.94	182.57±25.55	227.89±53.32	381.82±206.28	ND	101.97±35.12	410.14±416.74
Heptadecanoic acid, 15-methyl-, ethyl ester	29.26±16.13	30.23±13.13	27.04±3.36	164.2±56.41	123.99±13.86	49.18±6.33	1385.19±2164.52	187.32±55.14	54.94±7.24	123.97±8.86	157.01±36.36	247.73±84.44	ND	ND	ND
Phenylethyl alcohol	254.66±58.67	452.7±162.34	217.59±161.7	2515.13±415.07	927.16±223.11	626.7±132.98	2051.38±780.3	1673.97±510.23	625.07±26.13	961.78±124.58	1248.87±300.2	1889.96±588.16	402.87±55.18	562.58±147.81	677.43±102.6
9-Octadecenoic acid, ethyl ester	362.68±175.88	396.84±161.74	366.78±35.65	2022.1±410.84	1534.83±198.07	694.46±94.5	2648.96±1647.39	2246.93±706.3	565.24±115.7	1230.29±153.27	1715.17±372.89	2576.43±761.31	318.11±57.73	630.38±267.25	1345.38±1170.95
Nonanedioic	ND	ND	ND	14.15±12.48	19.05±2.21	8.9±0.55	31.71±11.96	25.65±5.92	13.49±1.11	17.45±2.19	18.48±2.91	29.55±10.83	7.11±0.34	8.7±2.45	9.18±2.22

acid,1,9-diethyl ester															
2-Methyl-4-heptanone	21.62±2.65	12.27±10.8	ND	ND	ND	ND	ND	ND	ND	ND	ND	ND	24.12±0.22	21.9±4.33	20.77±3.53
Hexanoic acid, butyl ester	ND	19.61±17.61	18.04±8	28.1±25.33	ND	ND	ND	ND	ND	ND	ND	ND	ND	ND	ND
Hexadecanoic acid, ethyl ester	163.67±255.72	759.13±279.99	636.99±59.69	2465.33±2094.24	2871.2±344.84	1175.34±127.11	5384.39±2591.56	5221.76±1545.49	1680.1±306.16	3472.63±340.61	4504.13±874.19	6906.93±2116.1	46.07±57.27	24.89±8.83	25.73±7.5
9,12-Octadecadienoic acid, ethyl ester	25.52±10.25	103.24±54.12	49.98±25.39	151.01±37.48	235.43±39.85	107.75±15.64	741.81±455.58	627.27±155.46	45.86±19.63	151.35±5.71	445.84±132.93	712.6±260.92	13.3±0.69	26.39±8.7	30.99±16.47
(E)-4-Decenoic acid, ethyl ester	ND	18±5.31	ND	ND	ND	25.71±2.56	ND	ND	42.65±3.54	ND	67.32±16.5	110.11±42.25	12.06±3.94	18.19±4.47	22.48±2.66
2-Methoxy-4-vinylphenol	15.53±4.16	ND	ND	33.86±5	19.06±2.39	9.85±1.71	17.52±15.81	ND	13.54±2.82	9.44±8.38	ND	ND	ND	ND	ND
Isobutyl alcohol	ND	ND	ND	79.9±17.34	22.26±5.61	15.04±2.64	57.03±23	44.11±14.72	15.82±0.6	38.76±3.8	33.01±5.58	58.61±13.97	12.96±2.44	17.65±6.23	22.81±6.78
Guaiacol	ND	ND	ND	36.1±7.69	ND	ND	32.02±11.29	28.49±7.82	ND	ND	23.31±4.85	37.45±10.82	7.4±1.04	10.7±2.75	12.55±2.06
2-Methoxy-4-methylphenol	ND	ND	ND	ND	ND	6.92±1.15	35.08±11.65	32.89±8.35	30.99±3.17	26.88±3.11	ND	ND	ND	ND	ND
2,4-Dihydroxybenzaldehyde	ND	ND	ND	ND	15.87±3.63	11.36±2.06	ND	ND	11.88±0.66	18.45±2.03	ND	ND	ND	ND	ND
2,4-Di-tert-butylphenol	8.08±9.09	3.22±3.02	11.84±5.56	27±25.46	23.54±4.67	11.42±3.73	29.44±15.67	20.98±3.59	31.42±12.6	37.4±4.8	47.76±16.49	64.55±12.52	ND	ND	ND
Lactic acid, ethyl ester	±28.67	28.73±13.7	41.47±4.25	98.33±12.63	57.38±18.52	30.3±7.14	132.7±56.73	131.28±45.61	41.97±3.6	87.71±9.6	121.43±39.28	ND	ND	129.76±37.59	168.67±31.33
2-Furanmethanol	7.69±6.66	14.01±15.21	23.29±2.75	48.96±8.37	24.71±6.31	13.76±2.82	36.33±17.56	34.13±10.99	ND	ND	ND	16.87±17.07	5.34±0.14	7.21±2.11	8.16±1.43
Furfural	17.54±15.19	ND	44.27±38.73	29.49±7.51	ND	ND	ND	ND	ND	ND	ND	ND	ND	ND	ND
Tetradecanoic acid, 13-methyl-, ethyl ester	20.21±16.03	34.9±9.65	23.87±3.2	91.59±15	80.69±13	41.65±3.73	320.9±119.56	290.41±65.1	85.18±5.76	77.03±6.83	231.62±38.57	394.25±129.18	27.18±5.2	38.31±11.2	52.22±27.85
Hexadecanoic acid, 15-methyl-, ethyl ester	11.95±6.9	10.42±4.64	ND	ND	35.16±5.21	14.74±1.44	53.25±37.49	65.01±16.16	14.59±2.39	30.02±2.3	19.29±2.36	69.14±35.6	ND	7.88±2.27	ND
9,12,15-Octadecatrienoic acid, ethyl ester	22.09±9.54	23.12±10.13	20.86±1.63	123.66±24.04	88.64±14.85	40.28±5.57	484.22±669.4	121.15±28.69	40.15±4.9	82.96±9.09	99±25.03	164.67±55.82	19.19±4.12	42.29±16.63	31.05±6
9.cis.,11.trans.-Octadecadienoic	ND	25.59±19.79	71.32±29.33	ND	25.04±2.75	10.4±2.59	71.14±29.66	105.97±101.1	20.14±5.86	68.97±6.5	36.31±23.01	80.44±13.36	ND	ND	ND

acid, ethyl ester															
9-Tetradecenoic acid, ethyl ester	ND	ND	ND	ND	13.05±2.17	7.28±0.76	18.81±21.79	14.06±12.82	11.21±2.15	9.32±8.09	16.97±3.03	15.65±14.98	5.78±0.66	9.85±3.01	24.78±25.21
9,12-Octadecadienoate, propyl ester	415.42±199.95	428.78±177.85	387.59±40.11	2273.96±448.34	1724.97±286.49	802.11±106.05	1358.56±1031.24	2513.06±704.4	801.07±161.35	1688.65±150.83	2185.74±500.6	3311.62±1003.33	410.71±84.72	834.77±317.59	734.81±201.94

Table S2. Volatile compounds of the fermented grain samples from round 2 based on gas chromatography-mass spectrometry analysis (μg/kg).

Flavor compounds	C2_0S	C2_0Z	C2_0X	C2_7S	C2_7Z	C2_7X	C2_14S	C2_14Z	C2_14X	C2_21S	C2_21Z	C2_21X	C2_CJS	C2_CJZ	C2_CJX
Benzyl alcohol	113.57±42.81	155.49±29.78	56.24±9.65	102.82±6.07	105.31±5.99	141.61±33.86	2.83±4.91	12.75±5.43	27.93±2.87	27.57±7.16	10.40±0.80	11.53±1.84	20.54±1.71	22.28±8.07	20.19±8.06
Benzaldehyde	207.15±68.73	284.36±34.65	74.81±18.68	114.99±18.83	199.36±8.47	274.51±64.56	4.18±3.7	7.8±3.27	15.59±1.49	20.36±4.66	10.07±0.69	10.65±1.3	25.6±3.06	20.26±6.58	24.35±8.25
Phenylacetic acid, ethyl ester	376.59±100.92	500.79±92.58	144.01±28.29	386.89±30.61	569.83±12.25	797.5±190.57	33.41±7.43	43.7±13.29	80.74±4.98	96.87±18.1	50.12±3.22	58.2±3.85	137.98±8.8	147.05±43.62	165.19±43.86
Acetic acid 2-phenylethyl ester	226.21±63.62	299.14±47.28	95.19±19.47	208.18±14	289.16±7.45	409.44±96.16	13.72±10.51	23.91±6.98	50.26±3.01	52.78±10.75	25.33±2.13	34.02±2.32	56.95±2.87	64.07±17.64	73.29±17.63
Pentanoic acid, 2-hydroxy-4-methyl-,ethyl ester	159.62±56.25	222.88±45.01	68.88±15.71	248.24±29.98	383.46±20.61	594.44±153.21	15.77±3.71	29.98±14.13	36.74±3.33	52.3±10.36	22.42±3.07	22.45±2.41	73.36±6.72	69.62±18.68	70.05±22.28
gamma-Nonanolactone	ND	ND	ND	ND	80.27±4.55	109.86±23.94	ND	ND	ND	ND	8.51±0.81	9.76±0.73	18.13±1.3	ND	19.32±6.21
Octanoic acid, ethyl ester	220.21±35.53	296.89±84.79	71.01±22.1	174.1±14.69	264.88±34.16	286.72±81.6	14.37±1.45	17.23±4.45	25.72±0.3	33.61±5.54	16.48±1.51	26.7±8.34	35.8±2	36.41±6.88	52.77±11.05
Dodecanoic acid, ethyl ester	142.22±24.6	165.26±29.59	62.92±25.02	180.81±21.54	257.23±27.11	349.29±37.02	18.28±5.58	22.23±4.98	36.2±3.25	50.2±11.81	22.56±4.07	26.89±1.71	45.72±1.81	38.2±13.61	41.23±10.27
(Z)-Ethyl heptadec-9-enoate	140.19±32.38	138.6±42.66	31.54±14.13	55.29±21.76	98.93±11.69	150.48±33.54	ND	ND	ND	ND	10.55±0.33	9.58±1.58	ND	5.47±4.9	ND
Phenol	ND	ND	10.48±18.15	ND	ND	ND	5.55±1.63	ND	ND	ND	ND	6.58±2.11	3.68±6.38	ND	4.96±8.59
Decanoic acid, ethyl ester	365.72±105.92	427.11±92.35	84.57±72.9	252.43±49.01	409.41±156.35	732.43±102.73	20.66±5.17	22.93±2.64	31.47±19.89	49.43±14.32	21.91±2.47	33.89±12.46	28.35±24.63	32.97±10.01	49.61±13.78
1-Hexanol	ND	34.45±29.88	10.79±9.63	41.07±2.7	62.69±3.74	85.86±22.19	1.9±1.69	1.02±1.77	ND	ND	ND	ND	10.71±0.99	ND	1.74±3.01

1-Octanol	ND	ND	9.44±16.35	15.55±26.93	35.7±31.64	ND	ND	3.44±3.24	5.44±4.72	ND	4.88±0.83	5.87±0.76	6.66±5.85	5.57±4.89	4.05±7.01
Decanal	50.44±43.84	ND	ND	9.44±16.35	ND	ND	ND	ND	ND	ND	ND	4.85±4.74	ND	ND	ND
Tetramethylpyrazine	67.18±28.81	87.64±15.99	28.96±5.86	49.18±3.41	59.89±5.42	80.08±18.26	2.82±2.6	5.67±2.23	11.18±1.61	11.57±2.94	ND	5.78±0.68	6.57±5.69	ND	12.4±4.59
2-Methoxy-5-methylphenol	ND	123.27±20.8	9.84±17.05	60.03±52.18	ND	155.62±44.8	5.52±4.85	ND	7.58±13.13	ND	ND	ND	ND	ND	ND
3-Phenylpropanol	40.83±10.31	57.17±6.99	17±2.84	41.13±3.18	47.93±1.46	65.91±12.52	3.68±1.14	5.48±1.89	11.71±0.96	11.55±2.9	4.56±0.5	5.28±0.54	9.42±0.32	9.96±3.83	8.24±2.83
4-Ethylphenol	77.18±67.59	169.04±15.46	17.17±29.74	110.76±13.98	138.33±8.19	192.79±34.43	7.49±2.32	10.32±4.05	21.97±1.51	22.81±6.1	8.73±1.14	9.83±0.92	19.45±1.54	18.84±7.14	18.94±6.42
Butanedioic acid, diethyl ester	158.91±41.27	230.47±56.81	76.72±17.7	197.78±21.33	247.38±21.1	335.06±87.56	19.42±6	25.82±9.82	37.15±32.28	66.53±17.36	25.72±2.85	28.45±2.57	67.31±6.6	82.37±34.01	84.47±29.39
Nonanoic acid, ethyl ester	37.16±32.79	80.73±20.53	11.89±10.75	53.25±7.46	74.14±19.66	84.96±18.33	6.66±4.24	1.77±3.06	5.16±4.48	10.92±3.25	6.46±0.58	7.66±2.02	10.93±0.82	6.22±5.73	11.62±1.87
3-Methyl-1-butanol	908.43±790.83	803.72±696.19	310.9±270	1207.06±74.95	1793.77±84.64	2846.6±678.73	83.16±19.42	121.98±34.62	ND	ND	87.23±12.97	89.06±11.45	271.38±29.89	148.78±48.66	198.1±73.32
Hexanoic acid, ethyl ester	278.83±394.3	53.54±47.16	23.71±6.11	45.15±5.22	69.13±0.78	75.85±17.52	3.3±2.96	1.99±3.44	ND	9.99±1.24	ND	8.12±2.96	13.33±0.8	10.26±2.14	13.37±3.49
Acetic acid isoamyl ester	ND	ND	ND	ND	88.06±5.68	111.72±27.47	ND	ND	ND	ND	ND	ND	11.34±0.96	ND	13.57±3.26
Tetradecanoic acid, ethyl ester	194.8±160.98	515.29±14.17	140.63±28.32	325.96±50.59	458.77±42.38	786.9±74.89	33.63±12.3	46.25±14.42	85.51±4.61	109.18±23.95	46.37±3.35	50.84±5.18	149.88±8.58	111.66±49.73	125.49±40.44
Nonanal	64.47±32.36	79.7±17.01	31.91±7.64	47.71±4.2	47.79±1.55	56.38±12.3	2.1±1.92	ND	ND	ND	ND	5.6±1.03	ND	ND	ND
Heptadecanoic acid, ethyl ester	135.78±135.36	80.41±5.63	27.26±14.57	57.77±13.16	80.11±9.23	107.3±14.71	5.1±4.43	8.33±2.85	16.36±4.58	20.56±3.93	11.07±1.26	10.35±2.09	49.3±3.52	11.3±11.28	36.01±18.24

Acetic acid ethyl ester	550.52±91.92	502.62±244.31	295.15±27.48	653±49.52	1255.65±116.4	1705.23±321.8	48.15±18.96	75.06±6.67	183.06±10.84	141.53±27.47	40.68±0.95	16.17±3.5	156.94±8.73	138.59±44.48	140.1±43.94
1-Nonanol	70.57±21.49	65.9±57.38	19.39±17.2	38.84±33.66	ND	ND	3.74±3.24	1.38±2.38	ND	9.27±1.57	ND	6.9±1.18	7.11±6.23	ND	6.91±6.11
Lactic acid isopentyl ester	ND	78.76±72.12	ND	ND	ND	ND	2.87±4.97	3.86±6.68	13.26±11.7	ND	ND	ND	19.51±11.21	13±11.93	28.38±24.25
Benzenepropanoic acid, ethyl ester	67.62±19.49	89.94±15.2	26.62±4.64	66.32±4.67	100.11±7.21	131.06±28.22	4.53±3.96	7.95±2.18	15.01±0.92	16.89±3.32	9±0.57	10.43±0.5	22.78±0.5	14.82±5.41	19.99±5.26
Hexadecanoic acid, propyl ester	ND	ND	ND	56.51±5.66	101.72±10.58	151.26±24.25	ND	8.95±4.94	ND	10.99±11.16	6.63±0.51	6.07±0.9	21.58±2.12	ND	12.17±5.08
2,3-Butanediol	279.9±61.49	399.43±57.74	61.99±15.95	135.66±22.11	206.39±21.81	276.9±75.27	12.03±5.1	17.74±8.1	54.36±21.48	45.24±14.6	14.47±1.4	13.78±2.73	37.93±10.74	32.1±13.79	40.73±17.29
4-Ethyl-2-methoxyphenol	160.29±44.79	212.05±29.13	66.15±10.73	157.61±13.71	225.29±5.19	322.39±69.71	13.52±3.2	16.86±5.51	35.06±2.72	38.65±9.53	16.37±0.82	16.67±1.36	41.99±2.42	35.91±12.62	35.34±11.1
4-Hydroxyphenylphosphonic acid	ND	ND	ND	61.82±9.35	64.34±0.87	118.18±68.06	ND	ND	ND	ND	ND	ND	10.46±9.07	ND	ND
5-Methyl-5-nonanol	ND	ND	ND	ND	ND	ND	4.57±0.34	5.26±0.22	ND	ND	4.48±0.66	ND	ND	ND	ND
1-Hexadecanol	ND	ND	ND	ND	ND	33.5±29.53	3.32±0.84	1.03±1.78	ND	ND	ND	ND	ND	ND	ND
Pentadecanoic acid, ethyl ester	150.87±38.36	175.18±14.02	41.77±36.18	127.64±20.91	182.13±18.14	111.36±95.07	17.23±16.99	18.17±6.09	34.53±1.26	28.51±27.06	19.01±1.61	19.81±3.21	52.88±2.55	66.51±38.33	48.36±18.72
3,4-Dimethoxytoluene	ND	ND	ND	34.47±3.61	ND	ND	1.75±1.55	5.01±1.72	8.89±0.82	8.63±1.84	ND	4.48±0.38	ND	6.3±5.69	ND
3-Furaldehyde	30.83±27.53	ND	21.01±3.84	ND	ND	ND	ND	ND	ND	ND	ND	ND	ND	ND	ND
Acetoin	188.49±74.78	259.5±39.08	ND	ND	49.67±43.09	104.61±25.17	ND	ND	ND	ND	ND	ND	ND	ND	ND
9-Hexadecenoic acid, ethyl ester	634.03±178.56	688.15±85.24	43.85±4.59	ND	606.02±78.79	842.53±107.16	7.38±3.3	50.45±17.48	21.9±1.11	126.21±38.49	51.89±2.77	51.67±7.78	138.66±9.06	22.43±11.93	134.11±51.71
Heptadecanoic acid, 15-methyl-, ethyl ester	330.19±122.16	406.87±57.31	138.28±12.86	189.1±167.6	393.71±50.34	489.5±78.55	26.85±12.58	45.69±19.1	ND	ND	42.74±4.74	43.43±7.64	115.61±10.13	114.13±65.59	116.29±51.64
1,2-Dimethoxy-4-ethyl-benzen	ND	57.2±9.35	18.34±3.38	63.47±5.2	ND	ND	5.36±1.12	9.37±3.1	14.56±2.05	15±3.08	7.17±0.6	8.58±0.62	16.79±1.59	15.61±5.53	10.94±2.83
Phenylethyl alcohol	4633.74±1652.83	6281.19±610.32	2278.21±520.14	2912.9±144.73	2945.71±160.1	4315.22±1040.91	272.06±86.34	368.65±147.02	839.23±89	780.54±206.93	280.14±36.99	403.24±58.66	513.94±39.78	548.37±192.74	736.87±283.12
9-Octadecenoic acid, ethyl ester	4293.64±1549.28	7058.45±4335.04	1489.44±149.68	2738.19±373.31	4162.52±544.45	5263.81±709.48	153.78±157.7	228.64±247.39	752.26±57	945.21±340.05	390.43±25.36	358.42±63.41	767.06±63.09	598.29±324.03	731.54±319.89
Nonanedioic acid,1,9-diethyl ester	ND	ND	9.06±7.85	39.1±6.09	58.8±7.72	85.2±18.14	ND	ND	ND	ND	5.45±0.25	5.67±0.85	13.41±0.85	7.19±7.91	ND

2-Methyl-4-heptanone	ND	ND	ND	ND	ND	ND	ND	ND	ND	ND	8.39±0.54	10.2±2.94	ND	5.39±4.7	3.06±5.31
Hexanoic acid, butyl ester	ND	ND	8.61±14.91	10.87±18.83	167.19±98.39	ND	ND	ND	ND	ND	ND	ND	ND	ND	ND
Hexadecanoic acid, ethyl ester	9373.47±2840.79	11010.6±1142.98	54±7.97	113.88±19.13	12856.69±1245.57	16970.54±2042.17	11.47±5.68	1250.66±471.48	1569.41±1335	3141.38±1043.63	1271.92±77.86	1315.2±246.96	3833.16±304.77	570.27±912.16	3496.85±1479.41
Acetic acid	ND	ND	ND	ND	ND	ND	ND	ND	ND	ND	ND	ND	ND	8.65±8.72	4.7±8.14
1-Pentanol	ND	ND	237.7±411.72	ND	ND	ND	ND	ND	157.2±137.34	190.14±164.67	ND	ND	ND	ND	ND
9,12-Octadecadienoic acid, ethyl ester	716.03±266.74	1035.96±134.74	72.96±2.14	136.99±15.85	950.37±126.06	1095.07±162.84	9.37±2.81	42.39±19.13	31.07±3.79	65.83±35.27	45.28±5.23	39.87±7.05	146.36±10.86	11.98±12.26	136.95±92.74
(E)-4-Decenoic acid, ethyl ester	ND	35.84±31.14	ND	21.48±18.79	54.25±6.7	70.88±13.79	2.56±2.26	4.24±0.84	5.13±4.44	5.35±4.72	5.23±0.51	6.33±0.99	10.42±0.68	9.7±3.35	11.09±2.72
Isobutyl alcohol	73.62±21.92	83.62±21.96	37.61±10.08	85.96±4.71	130.72±8.99	227.47±56.8	6.62±1.84	9.11±2.67	20.37±2.37	21.62±3.56	7.66±1.48	3.95±3.42	20.82±0.91	14.17±5.28	15.87±5.73
2-Methoxy-4-methylphenol	70.23±62.69	ND	ND	ND	108.03±8.68	ND	ND	ND	ND	ND	7.05±0.94	8.07±0.53	16.73±0.97	14.19±4.09	15.31±4.17
Benzoic acid, ethyl ester	ND	ND	ND	21.01±18.26	52.28±0.73	67.79±15.17	ND	0.88±1.52	ND	ND	ND	ND	5.93±5.16	ND	1.76±3.04
2,4-Di-tert-butylphenol	78.38±46.71	71.78±17.25	24.52±5.4	ND	56.69±12.67	60.42±3.08	3.99±0.85	0.79±1.37	ND	ND	6.26±5.53	ND	9.6±8.74	7.5±6.66	9.21±15.95
Lactic acid, ethyl ester	399.09±154.61	540.94±90.41	202.8±42.78	762.53±425.6	758.74±657.22	1203.93±1132.45	53.46±17.88	83.03±38.99	147.95±15.52	127.15±121.04	69.29±10.81	69.76±13.22	223.37±21.36	377.14±137.28	310.72±128.88
2-Furanmethanol	ND	ND	ND	ND	ND	ND	4.2±3.7	7±2.47	11.36±0.7	12.27±3.17	ND	5.9±1.19	12.12±0.96	11.45±3.67	20.5±9.1
Furfural	ND	85.45±26.69	ND	143.36±21.86	ND	420.99±101.12	ND	ND	ND	14.61±4.71	ND	ND	12.23±10.62	ND	ND
Tetradecanoic acid, 13-methyl-, ethyl ester	564.22±172.28	610.56±20.42	25.68±44.48	218.7±30.89	642.82±43.46	901.8±237.38	26.36±11.19	31.48±10.31	55.29±2.16	74.27±19.13	31.13±3.48	30.31±3.57	133.84±9.02	45.31±33.92	112.95±40.4

Hexadecanoic acid, 15-methyl-, ethyl ester	104.73±50.44	143.23±45.25	19.38±22.59	ND	139.03±40.56	177.45±53.18	2.47±2.26	8.39±4.32	7.78±0.36	24.29±14.85	4.48±0.47	3.24±2.85	12.14±1.37	5.32±5.25	20.15±17.97
Flavor compounds	2L0S	2L0Z	2L0X	2L7S	2L7Z	2L7X	2L14S	2L14Z	2L14X	2L22S	2L22Z	2L22X	2LC30S	2LC30Z	2LC30X
9,12,15-Octadecatrienoic acid, ethyl ester	470.82±171.75	567.02±96.75	140.8±18.95	266±41.05	342.5±275.71	579.23±131.37	20.55±10.11	32.8±14.28	75.55±11.87	95.66±35.92	37.83±2.97	37.05±3.86	77.61±2.41	71.11±37.71	89.47±4.23
9,12-Hexadecadienoate, ethyl ester	45.35±23.84	31.68±27.44	4.59±7.95	ND	ND	ND	ND	ND	ND	ND	ND	ND	ND	ND	ND
9.cis.,11.trans.-Octadecadienoic acid, ethyl ester	191.52±243.97	89.67±111.07	ND	ND	156.46±56.25	154.57±224.87	ND	ND	ND	ND	15.17±2.12	13.12±2.76	32.54±5.17	ND	45.58±1.14
9,12-Octadecadienoate, propyl ester	6683.26±2206.2	7475.9±1252.83	2556.89±258.72	4963.84±669.58	6740.33±905.03	8871.39±943.67	362.72±185.47	597.84±248.5	1224.99±86.36	1569.71±542.45	700.22±38.48	657.6±115.66	1365.73±104.82	1168.38±630.83	1239.29±534.94

Table S3. Volatile compounds of the fermented grain samples from round 3 based on gas chromatography-mass spectrometry analysis (μg/kg).

Flavor compounds	C3_0S	C3_0Z	C3_0X	C3_7S	C3_7Z	C3_7X	C3_14S	C3_14Z	C3_14X	C3_21S	C3_21Z	C3_21X	C3_CJS	C3_CJZ	C3_CJX
Benzyl alcohol	69.9±8.19	79.08±7.42	ND	ND	ND	ND	ND	ND	ND	60.28±22.77	563.81±592.14	52.3±0.7	107.47±38.59	ND	131.21±112.25
Benzaldehyde	66.48±7.4	70.64±5.98	ND	102.54±18.46	62.6±88.53	96.5±2.98	416.43±388.41	198.22±10.31	263.71±60.07	265.23±50.54	794.01±545.12	ND	410.89±170.76	347.85±171.65	239.94±74.42
Phenylacetic acid, ethyl ester	356.51±19.85	428.83±30.25	4183.15±1091.08	843.34±131.4	937.86±223.99	810.19±25.11	3559.91±3320.67	1559.28±122.81	2018.35±347.51	723.7±638.2	ND	716.89±0.86	1480.86±346.06	1044.59±737.61	406.23±642.84
Acetic acid 2-phenylethyl ester	244.87±8.93	308.78±19.94	1043.62±238.37	612.82±95.18	745.12±154.78	694.01±14.88	2517.41±2390.84	861.81±746.38	1086.78±979.18	758.46±226.52	6227.71±6768.32	ND	898.5±227.3	612.06±449.89	723.41±110.12
Pentanoic acid, 2-hydroxy-4-methyl-, ethyl ester	180.15±20.12	200.96±9.8	1611.83±340.71	408.35±56.49	ND	ND	ND	ND	ND	ND	ND	ND	ND	ND	ND
gamma-Nonanolactone	38.9±1.19	42.76±3.61	281.65±86.69	77.54±9.38	ND	ND	ND	ND	ND	ND	ND	ND	ND	ND	ND
Butanoic acid, ethyl ester	34.33±3.62	43.27±0.82	4344.08±1002.64	65.07±7.4	ND	ND	ND	ND	ND	ND	526.85±602.24	29.79±0.88	29.69±25.78	ND	30.47±3.82
Octanoic acid, ethyl ester	226.72±16.38	254.6±5.91	1977.96±541.16	461.44±80.7	534.2±150.63	415.19±6.98	1884.81±1800.6	738.05±144.65	1096.73±186.41	2185.53±281.67	4119.31±1192.74	369.11±0.83	1735.01±147.94	788.04±443.62	1385.01±494.41
Dodecanoic acid, ethyl ester	144.5±7.84	164.57±10.93	854.54±227.42	323.81±55.85	321.63±99.01	298.46±19.89	1153.82±1062.39	505.5±63.44	679.85±129.98	316.78±112.66	2754.23±3075.75	ND	206.51±179.04	234.54±159.78	207.16±162.07
Hexanoic acid, methyl ester	ND	ND	ND	ND	ND	ND	ND	ND	ND	ND	2819.56±3782.2	ND	371.72±22.55	ND	300.35±133.58
(Z)-Ethyl heptadec-9-enoate	ND	ND	ND	ND	40.14±8.27	44.32±0.17	161.89±157.07	129.4±111.1	ND	18.29±16.93	ND	ND	ND	ND	ND
Phenol	25.69±2.45	29.37±2.52	102.2±25.99	48.86±7.88	22.41±31.69	51.05±1.66	ND	ND	ND	ND	ND	36.94±0.99	ND	ND	ND
Decanoic acid, ethyl ester	258.06±26.79	266.64±7.85	1403.97±356.81	524.18±120.58	514.46±163.58	375.41±10.78	1754.52±1647.45	803.12±186.68	1053.37±158.85	405.67±390.88	1812.39±795.34	390.54±1.02	380.76±96.92	273.1±183.55	384.53±29.27
2-Heptanone	ND	ND	ND	ND	ND	ND	ND	ND	ND	356.55±25.	3637.71±466	ND	343.1±304.	104.04±31.	374.5±161.

										93	5.94		38	35	55
Octanoic acid, methyl ester	ND	ND	ND	ND	ND	ND	ND	ND	ND	122.78±129.69	412.66±57.15	ND	204.84±4.21	74.01±42.19	156.21±74.45
1-Hexanol	22.55±2.61	24.05±1.89	1164.14±319.97	40.87±5.89	49.52±11.63	45.21±0.04	164.2±149.51	76.61±2.54	109.36±20.56	126.35±180.38	329.57±276.28	44.78±0.94	75.9±26.16	42.32±29.72	47.36±12.77
1-Octanol	ND	40.3±1.49	ND	ND	ND	ND	ND	ND	114.64±20.8	ND	ND	ND	ND	ND	ND
Tetramethylpyrazine	61.96±3.74	47.03±2.4	194.86±45.79	62.61±13.65	85.62±11.85	55.29±4.26	99.75±16.47	125.23±73.16	184.31±146.92	83.85±60.2	157.61±115.59	51.79±0.6	74.01±51.82	33.38±14.93	39.61±22.99
3-Phenylpropanol	22.19±1.74	25.14±2.34	ND	39.37±4.09	42.73±6.33	44.01±1.95	150.65±138.77	78.12±5.11	95.24±17.99	31.32±10.55	268.99±284.39	ND	44.44±13.79	30.62±23.95	30±4.11
Butanedioic acid, diethyl ester	213.16±22.44	257.74±23.02	1698.81±383.7	506.74±64.39	574.09±152.25	477.17±13.2	1946.68±1854.73	872.68±27.51	1134.01±193.33	409.68±146.97	1304.51±816.54	348.6±1.54	428.05±419.64	440.24±336.54	344.5±95.41
3-Methyl-1-butanol	398.27±54.87	ND	1588.42±424.4	796.81±102.86	889.66±185.71	855.34±24.66	3192.39±2993.21	1555.93±86.45	1998.52±377.55	589.28±213.12	4623.38±4613.17	663.53±0.88	872.28±339.86	611.7±495.65	500.51±81.32
Hexanoic acid, ethyl ester	285.36±25.96	300.31±25.14	21371.58±5590.01	431.44±79.69	722.48±146.31	346.46±42.13	1930.28±1859.65	715.74±141.48	1203.12±351.45	2580.4±992.2	ND	168.4±0.71	2200.32±1943.91	820.51±433.79	2986.46±1536.2
Acetic acid isoamyl ester	63.26±5.9	80.38±1.32	ND	167.02±26.28	ND	ND	ND	ND	ND	ND	ND	ND	ND	ND	ND
Tetradecanoic acid, ethyl ester	143.6±27.92	163.67±27.27	854.85±244.13	356.68±59.85	392.69±75.78	120.26±6.79	1461.59±1291.34	636.68±61.91	851.89±117.74	ND	3606.06±3698.01	286.31±0.95	493.55±147.29	104.02±28.67	369.61±50.17
3-(Methylthio)-propanoic acid ethyl ester	27.44±8.1	27.6±10.65	236.4±59.34	91.19±15.1	99.9±27.82	97.3±0.29	434.15±401.93	186.86±14.49	255.99±39.78	77.15±39.72	51.64±18.35	81.57±14.69	72.69±15.41	58.98±42.18	52.18±8.94
Heptadecanoic acid, ethyl ester	ND	16.96±3.26	ND	48.34±9.63	46.42±23.93	40.94±0.12	129.95±94.53	91.73±11.99	82.4±4.7	38.6±16.09	352.64±349.31	56.47±1.2	ND	53.75±48.24	35.39±7.85
Acetic acid benzyl ester	ND	ND	ND	ND	30.23±3.66	26.5±0.93	ND	14.95±25.9	ND	ND	ND	ND	ND	ND	ND
Acetic acid ethyl ester	486.6±69.21	602.16±7.33	1292.74±300.25	1310.19±98.29	1129.44±88.83	1271.29±130.42	2840.27±2163.89	1658.74±221.77	2104.43±1221.43	636.86±387.77	5751.2±6627.71	391.25±0.41	654.88±285.45	634.02±358.25	738.47±127.36
1-Nonanol	22.55±1.91	ND	ND	51.41±10.31	64.76±17.11	50.81±1.12	181.37±165.28	46.76±40.76	127.28±35.84	41.72±39.91	ND	33.25±0.57	ND	ND	ND
2,3-Butanediol	114.74±60.52	84.92±13.52	141.56±29.29	144.1±18.45	129.97±66.21	206.77±55.55	781±826.41	529.83±161.63	830.14±74	105.26±75.62	366.76±240.92	165.32±1.3	171.87±73.64	122.44±84.56	257.36±86.11
Lactic acid	ND	ND	ND	ND	170.88±35.	ND	ND	86.87±150.4	ND	120.39±16	117.33±61.13	229.73±1	75.28±20.9	70.83±4.95	42.41±7.06

isopentyl ester					22			7		9.24		.54	3		
Benzenepropanoic acid, ethyl ester	38.33±5.98	49.82±3.69	906.82±274.98	101.05±18.68	110.77±27.49	89.38±2.95	345.87±326.84	149.72±14.68	197.54±34.15	113.76±32.31	883.29±957.51	ND	114.38±27.95	84.3±57.26	75.61±35.01
á-Ethylphenethyl alcohol	19.36±1.44	23.18±2.31	ND	40.37±6.77	41.12±9.71	42.28±1.79	ND	66.6±9.11	87.26±14.46	ND	ND	ND	ND	ND	ND
Hexanoic acid 3-methylbutyl ester	ND	ND	1153.07±405.1	ND	ND	ND	ND	ND	ND	54.07±46.87	168.4±67.24	ND	70.43±16.2	ND	46.8±14
4-Ethyl-2-methoxyphenol	41.72±2.54	50.38±3.89	486.81±151.07	91.73±12.3	51.89±73.38	84.43±1.02	306.23±286.68	136.41±12.99	125.48±113.15	ND	ND	ND	ND	ND	ND
Pentadecanoic acid, ethyl ester	62.78±9.77	72.86±10.33	401.22±116.48	145.18±23.23	160.39±28.1	ND	ND	77.63±134.46	ND	95.18±98.14	ND	ND	ND	ND	91.56±14
(R)-(-)-1,2-Propanediol	28.57±15.53	14.71±2.27	ND	33.62±5.82	20.02±28.31	85.17±52.75	287.56±351.75	109.62±47.93	141.01±125.7	ND	639.44±763.09	ND	50.83±45.25	ND	33.01±57.17
9-Hexadecenoic acid, ethyl ester	131.12±33.42	140.22±29.86	608.99±181.72	293.32±55.29	359.19±49.83	405.01±16.7	1268.99±1202.86	605.22±73.45	799.48±78.87	49.45±52.29	1420.06±963.36	ND	188.98±153.43	93.53±31.11	64.16±12.26
(Z)-Ethyl pentadec-9-enoate	ND	ND	ND	ND	40.87±10.63	44.26±2.05	143.8±137.52	61.49±8.8	ND	ND	ND	25.59±1.02	ND	ND	ND
Heptadecanoic acid, 15-methyl-, ethyl ester	ND	47.39±13.02	273.02±81.24	121.17±23.09	ND	149.35±7.89	ND	2616.46±2086.46	333.63±48.94	ND	ND	ND	ND	ND	ND
1,2-Dimethoxy-4-ethyl-benzen	26.68±1.72	26.92±2.24	156.17±49.02	46.88±5.67	30.55±43.2	52.4±1.19	177.19±169.34	90.8±9.39	116.57±18.84	ND	ND	ND	ND	ND	ND
Phenylethyl alcohol	1921.72±220.12	2303.11±212.94	6356.53±1513.29	3582.35±508.88	3869.2±910.35	3927.6±143.11	14390.04±13646.38	7265.07±581.91	9295.69±1478.23	2650.25±970.26	8719.14±5862.14	2176.09±0.93	3769.23±1325.12	2560.37±2018.33	1724.54±1428
9-Octadecenoic acid, ethyl ester	733.12±248.8	835.07±214.53	4504.94±1300.16	1833.91±386.11	2057.74±516.09	2499.65±49.95	7495.93±7135.7	5943.62±2387.09	4652.36±521.27	1418.83±1495.9	ND	ND	ND	ND	ND
Hexanoic acid, butyl ester	ND	ND	1542.45±518.62	ND	ND	ND	ND	ND	ND	478.85±535.11	5642.07±7466.16	ND	648.33±104.75	187.19±92.31	473.03±258.74

Hexadecanoic acid, ethyl ester	2199.66±580.57	2630.23±548.03	14742.95±3833.64	6375.69±1131.17	6858.76±1471.99	7818.57±524.9	24830.3±23582.33	11384.33±1308.53	14937.13±1490.33	167.92±67.22	24107.82±16596.46	7526.8±1.68	4631.18±7776.53	745.09±978.63	4347.48±3753.94
Hexanoic acid, hexyl ester	ND	ND	507.82±215.04	ND	ND	ND	ND	ND	ND	212.47±34.03	944.56±1226.51	ND	48.87±44.06	26.18±16.69	46.34±22.49
Acetic acid	ND	23.07±0.79	ND	ND	ND	101.82±3.07	ND	ND	ND	ND	ND	ND	ND	ND	ND
Lactic acid, ethyl ester	417.4±58.98	493.67±44.62	4806.69±1316.02	1094.72±162.92	512.81±725.23	1199.82±2.5	6734.86±6417.26	3414.62±197.37	3296.45±2892.13	ND	ND	2004.75±1.73	ND	ND	ND
9,12-Octadecadienoic acid, ethyl ester	47.83±12.73	1893.69±435.01	294.75±81.7	139.64±21.86	150.57±31.56	181.65±2.65	ND	93.05±161.16	404.58±80.83	5051.28±2338.8	3804.23±4352.99	35.16±0.63	1284.66±2022.19	1700.84±2352.37	33.39±29.06
(E)-4-Decenoic acid, ethyl ester	46.88±3.77	44.99±0.82	275.82±96.37	92.69±15	31.6±44.69	63.18±1.64	ND	93.5±81.28	173.31±27.61	ND	ND	ND	ND	ND	ND
2-Methoxy-4-vinylphenol	20.57±5.94	ND	ND	29.48±4.11	47.43±5.48	37.81±0.84	113.56±109.44	54.86±8.96	65.3±12.98	35.65±10.17	327.05±392.57	28.46±0.96	ND	25.89±19.28	16.15±14.72
Isobutyl alcohol	22.25±2.35	22.44±1.19	118.94±40.34	38.41±6.86	47.75±1.77	41.77±3.22	174.27±166.32	84.21±9.53	108.52±19.84	35.75±11.49	294.72±293	27.19±0.66	65.64±26.99	37.57±27.59	31.76±5.98
Diethyl pentanedioate	8.2±7.16	15.82±0.86	ND	29.29±5.38	35.91±5.71	27.6±1.84	112.58±104.81	51.15±6.89	66.45±11.82	ND	ND	ND	ND	ND	ND
2-Nonanone	12.62±1.09	ND	ND	ND	18.45±26.09	30.84±1.49	131.97±122.06	58.44±1.8	84.85±8.66	ND	ND	ND	ND	ND	ND
1,2-Dimethoxybenzene	15.4±4.51	14.4±1.32	ND	ND	20.96±29.65	ND	96.67±88.53	ND	ND	ND	ND	ND	ND	ND	ND
2-Methoxy-4-methylphenol	24.3±1.69	25.62±2.16	414.76±122.99	46.6±7.96	ND	ND	ND	ND	ND	ND	ND	ND	ND	ND	ND
2,4-Di-tert-butylphenol	23.23±3.57	20.87±4.19	ND	34.81±6.15	16.55±23.41	ND	ND	ND	ND	ND	ND	ND	ND	ND	ND
2-Furanmethanol	67.4±16.63	64.79±5.87	363.26±73.72	105.69±16.65	101.66±31.11	100.15±2.11	358.31±336.03	154.69±13.77	210.36±33.08	ND	ND	ND	ND	ND	ND
Tetradecanoic acid, 13-methyl-, ethyl ester	102.51±22.11	121.01±21.75	371.5±95.29	239.47±42.31	263.35±59.74	271.41±8.97	915.35±857.77	383.63±42.66	514.32±80.27	257.39±98.21	1981.39±1942.69	83.96±0.31	293.06±97.89	193.09±154.52	197.75±41.8
Hexadecanoic	20.23±5.59	ND	ND	ND	41.88±2.39	61.89±1.73	191.97±205.7	15.35±26.59	117.56±14.5	54.48±32.8	189.82±200.1	ND	76.12±31.8	ND	36.73±32.6

acid, 15-methyl-, ethyl ester							2		3	1			3		3
9,12,15-Octadecatrienoic acid, ethyl ester	81.29±23.81	98.88±23.21	3669.84±5773.37	200.2±26.7	184.37±50.86	248.31±4.23	796.99±759.02	327.61±19.78	493.35±65	143.89±110.3	1386.3±1346.78	120.65±0.9	241.96±134.45	187.49±151.41	164.98±42.91
9.cis.,11.trans.-Octadecadienoic acid, ethyl ester	ND	23.45±3.84	125.32±47.81	50.95±11.22	ND	ND	ND	ND	ND	ND	ND	ND	ND	ND	ND
9-Tetradecenoic acid, ethyl ester	ND	13.91±1.83	ND	33.6±4.61	35.06±9.53	ND	98.6±90.32	67.97±10.19	90.72±12.74	46.49±12.95	602.62±496.43	ND	49.89±11.71	40.17±29.74	43.66±6.49
Methoxyacetic acid, 3-methylbutyl ester	ND	58.82±2	524.53±98.4	117.51±19.56	ND	ND	ND	ND	ND	ND	ND	ND	ND	ND	ND
9,12-Octadecadienoate, propyl ester	1526.27±441.04	ND	7956.41±2141.27	3694.82±839.23	3723.67±1137.82	4745.84±88.37	14460.61±13817.96	6920.99±878.74	8900.88±1200.78	70.03±25	203.73±141.9	2908.74±1.11	2726.89±4628.21	436.27±626.47	35.42±34.21
